# Evaluation of intravascular irradiation of blood in children with sleep bruxism: Study protocol for a randomized controlled clinical trial

**DOI:** 10.1097/MD.0000000000031230

**Published:** 2022-11-04

**Authors:** Natalia Osorio Viarengo, Marcela Leticia Leal Gonçalves, Laura Hermida Bruno, Ana Laura Fossati, María Roxana Ferreira Sertaje, Elaine Marcilio Santos, Ana Paula Taboada Sobral, Raquel Agnelli Mesquita-Ferrari, Kristianne Porta Santos Fernandes, Anna Carolina Ratto Tempestini Horliana, Lara Jansiski Motta, Sandra Kalil Bussadori

**Affiliations:** a Universidad Católica del Uruguay (UCU), Montevideo, Uruguay, South America; b Post Graduation Program in Biophotonics Applied to Health Sciences, Universidade Nove de Julho, São Paulo, SP, Brazil; c Postgraduation Program in Health and Environment, Universidade Metropolitana de Santos, Santos, SP, Brazil.

**Keywords:** bruxism, intravascular irradiation of blood, myofunctional therapy, oximetry, photobiomodulation

## Abstract

The objective of this study will be to evaluate the effect of intravascular irradiation of blood (ILIB) combined with myofunctional exercises for sleep bruxism in pediatrics.

**Methods:**

This study will be a randomized controlled clinical trial. A triage of individuals between 4 and 17 years old with a diagnosis of sleep bruxism will be carried out at the clinic of the Catholic University of Uruguay, and in a private office referred by different private care centers. The selected participants will be evaluated before and after treatment by means of questionnaires on bruxism, sleep quality and nocturnal oxygen saturation measurement. For this, 46 patients with sleep bruxism will be recruited, who will be divided into 2 groups: control group (CG), which will undergo an application of placebo ILIB and an orofacial myofunctional therapy (MFT) exercise protocol; and na ILIB group, which will carry out an active application of ILIB and an exercise protocol, this being once a week for 8 weeks. The laser treatment (808 nm) will be performed twice a week for 8 weeks. The values will be tested for normality by the Kolmogorov–Smirnov test. For the comparison between the groups, *t* test will be carried out, considering a level of significance of 0.5% (*P* < .05).

**Discussion:**

Although local photobiomodulation (PBM), acupuncture PBM and physiotherapy have been studied in the treatment of bruxism, this is the first study to evaluate the effect of ILIB combined with myofunctional exercises for sleep bruxism in pediatrics.

## 1. Introduction

Bruxism is a repetitive activity that involves clenching or grinding the teeth during sleep or wakefulness.^[1,2]^ In 2013, in an international consensus, it was defined as “a repetitive masticatory muscular activity, with clenching or grinding of the teeth and/or by the fixation (bracing) or thrust (thrusting) of the jaw.” In 2018, a new update was made, in which the thrust and fixation mandibular movements are defined in detail.^[3]^ The grinding is generally audible and triggers discomfort or side effects for those who present it. This condition is commonly seen in children and adolescents.^[4]^ If it is not identified and addressed in time, it can bring negative consequences such as tooth wear, periodontal disease, hypertrophy of the masticatory muscles, headache, limitation of mouth opening, muscle pain and temporomandibular disorders (TMD) and, in many cases, decrease in academic performance of the child or adolescent.^[5]^

Regarding the etiology of bruxism, there is consensus in the literature that bruxism has a multifactorial etiology.^[6]^ The genetic causes related to a possible polymorphism, anxiety, some personality traits, and endocrine alterations associated with stress, among others, are considered. It has also been related to occlusal factors, malocclusion, nutritional deficiencies, respiratory problems (apneas, snoring), allergies, intestinal parasites, oral habits, changes in neurotransmitters such as dopamine and sleep quality.^[7]^

When it comes to the diagnosis of bruxism, there are controversies. Currently, it is considered that only polysomnography can give a definitive diagnosis, but this exam has a high cost.^[8]^ Polysomnography allows simultaneous monitoring of electroencephalographic, electrocardiographic, electromyographic sleep, and respiratory signals during sleep.^[9]^ Self-questionnaires are usually used (in the case of pediatrics it is completed by the parents or the referent), based on the criteria of the American Association of Sleep Medicine. The prevalence is highly controversial. In the analysis of systematic reviews, a prevalence of 22% to 31% for sleep bruxism in adults was shown.^[10]^

Regarding therapeutics, they are mostly palliative, addressing the symptom, but without providing a solution. A systematic review suggests that the evidence is still scarce in relation to drugs.^[11]^ Botulinum toxin protects orofacial structures (teeth, jaw muscles, temporomandibular joint) reducing the damage caused by bruxism, generating a decrease in pain and symptoms related to excessive muscle contraction.^[12]^ Occlusal devices, such as splints, are used to protect teeth from the damage caused by clenching and grinding during bruxism.^[13]^ Photobiomodulation (PBM) is a very promising, painless and low-cost option; capable of stimulating or inhibiting cellular processes.^[14]^ Intravascular Irradiation of Blood (ILIB), a systemic modality of PBM, can be used as a complementary therapy to other therapies.^[15]^ It emits energy that activates neurohumoral regulation and synchronization and cell modulation with antioxidant, metabolic, immunological, antispasmodic, sedative, healing, analgesic, anti-inflammatory and blood circulation-increasing effects.^[16,17]^ Orofacial myofunctional therapy (MFT) is a set of techniques and procedures whose objective is to promote changes in muscle patterns and orofacial functions through isotonic and isometric exercises with the orofacial and oropharyngeal muscles supported by the functions of breathing, chewing, swallowing and talk.^[18]^ Orofacial MFT and systemic low intensity laser administration are options to more traditional therapies.

The objective of this study is to determine the efficacy of ILIB in sleep bruxism, in combination with oral MFT. It also aims to evaluate the relationship between oxygenation and bruxism during sleep and to determine the prevalence of nocturnal hypoxemia and its association with daytime oral breathing.

## 2. Methods/design

### 2.1. Study design

This is the first version of a study protocol for a randomized, controlled, parallel group, single blind, clinical trial, with a control group (CG) and na active comparator group, in which 46 individuals with a diagnosis of sleep bruxism will participate. The project was evaluated and approved by the Research Ethics Committee of *Universidad Católica del Uruguay* (process number 220211) and was registered in the Clinical Trials platform (https://clinicaltrials.gov/) with the number NCT05301452, first published on March 16, 2022 and last updated on August 4, 2022. Recruitment and treatments will be performed at the dentistry clinic of Universidad Católica del Uruguay clinics, during the period from September 1, 2022 to December 1, 2022.

**Figure 1.**
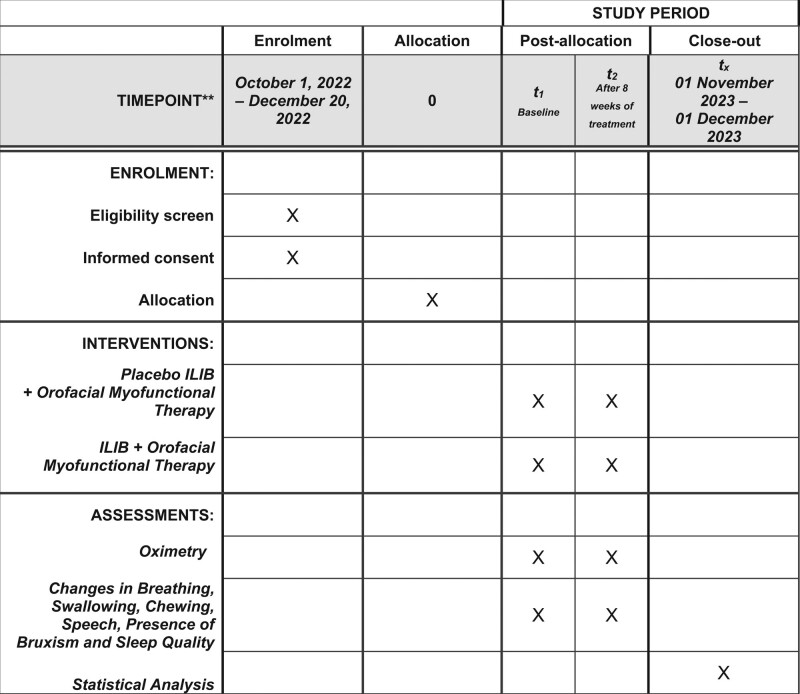
SPIRIT figure as recommended by 2013 SPIRIT statement. SPIRIT = standard protocol items: recommendations for interventional trials.

This protocol is in accordance with the 2013 SPIRIT (Standard Protocol Items: Recommendations for Interventional Trials) Statement. The SPIRIT checklist can be found as an additional file and Figure 1 is the SPIRIT figure.

### 2.2. Participants

A triage of individuals between 4 and 17 years old, diagnosed with sleep bruxism, will be carried out at the dentistry clinic of the Catholic University of Uruguay. Patients will already be in treatment for other conditions at the clinic, what will facilitate recruitment. It is believed that the need for treatment of bruxism before major pain symptoms or dental wear will help to improve adherence to the trial.

Inclusion criteria:

- Children in the mixed dentition phase (permanent incisors and first molars erupted);
- Or adolescents with an established permanent dentition.

Exclusion criteria:

- People with dental caries;
- Those taking medications, such as inflammatory agents, muscle relaxants, corticosteroids, anticonvulsants, and antidepressants;
- Those with chronic diseases that affect muscles or motor coordination, and those who do not cooperate during the evaluation will be excluded from the study;
- Children with cerebral palsy;
- Patients whose guardians do not sign the informed consent or the participant who does not sign the assent form.

### 2.3. Experimental groups

Forty-six patients with sleep bruxism will be recruited and divided into 2 groups:

- CG which will undergo an application of placebo ILIB and an orofacial MFT exercise protocol;

- ILIB group, which will carry out an active application of ILIB and an orofacial MFT exercise protocol, this being once a week for 4 weeks.

### 2.4. Sample calculation

**Figure 2.**
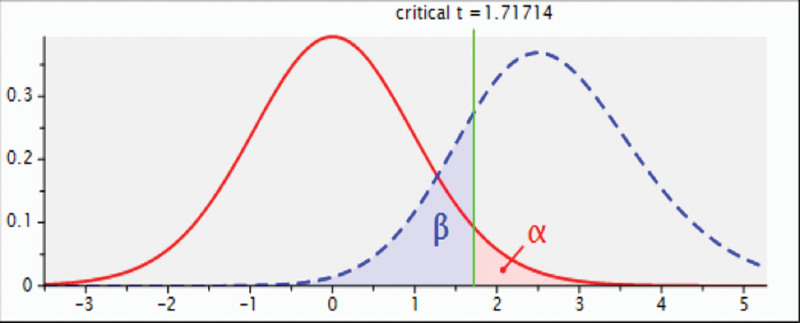
Sample size calculation. Analysis: A priori: Compute required sample size; Input: Tail(s) = One; Effect size dz = 0.5373031; *α* err prob = 0.05; Power (1-*β* err prob) = 0.8; Output: Noncentrality parameter *δ* = 2.576815; Critical t = 1.7171444; Df = 22; Total sample size = 23; Actual power = 0.8027361.^[19]^

The sample was calculated to detect a significant difference between PBM and MFT with 80% power and a significance level of 5%, considering the oxygen saturation index. Based on the study by Aarab et al^[19]^ to detect a difference in saturation index, with an effect size of 0.53, the minimum number per group should be 23 participants per group (Fig. 2).

### 2.5. Randomization

To randomly distribute the individuals in the experimental groups, a random sequence generator (https://www.randomizer.org/tutorial/) will be used. Opaque envelopes will be identified with each number and inside it a sheet containing the information of the corresponding experimental group will be inserted. The envelopes will be sealed and kept in a safe place until the time of the procedures. The generation of the sequence and the preparation of the envelopes will be performed by a person who is not involved in the study.

### 2.6. Blinding

The participants will not know whether they belong to the placebo/CG, or the active PBM group, seeing as the laser application will be simulated. Unblinding is not permissible.

### 2.7. Interventions

#### 2.7.1. ILIB protocol.

The laser treatment will be performed by a speech therapist, using the wavelength of 660 nm. It will be done once a week for 4 weeks, in 20-minute sessions. In the CG, the ILIB procedure will be simulated, but with the laser turned off.

#### 2.7.2. Orofacial MFT.

MFT will be performed by a speech therapist specialized in the area. It will consist of 1 weekly session, where the functions of breathing, chewing and swallowing will be evaluated and addressed.

The following exercises will be indicated: nasal hygiene, inflated cheeks with the tip of the tongue on the palate, lingual sweep, tongue thrust against the palate, tongue touch on the last molars, chewing of soft solids and bolus assembly (20 minutes therapy session).

## 3. Outcome measures

All participants will undergo a parent or referent bruxism questionnaire, infant sleep quality questionnaire, nocturnal oximetry, and clinical assessment of orofacial functions at baseline and after 4 weeks of treatment. Patient retention and complete follow-ups are expected due to the treatment of bruxism symptoms.

### 3.1. Oximetry

Recorded sleep oximetry monitoring has high specificity and sensitivity for the diagnosis of respiratory disorders in children. This is the primary outcome of the study.

A portable pulse oximeter that includes Masimo SET® Measure-through Motion and Low Perfusion™ pulse oximetry under motion and low perfusion conditions. Nocturnal oximetry will be performed prior to treatment and post treatment. The parents or guardian of the minor will be trained.

### 3.2. Breathing

A clinical evaluation will be carried out by a speech therapist specialized in the area of Orofacial Motricity, to check for possible improvement in breathing. It is the respiratory characterization from the taking of air, which explains the anatomical and topographical situation of its entrance to the respiratory system (respiratory mode), to the thoracoabdominal movement zone that integrates inspiration and expiration. It is supported by the observation with the aid of a Glatzel’s mirror to anatomically determine the upper airway status and its functional correlation.

### 3.3. Swallowing

A clinical evaluation will be carried out by a speech therapist specialized in the area of Orofacial Motricity, to check for possible improvement in swallowing. For this, the Payne’s test, which assesses tongue posture both at rest and in function (swallowing), will be used. From instruments such as liquid fluorescein applied to the apex lingual and lateral borders, and Payne’s lamp that verifies the vestige of the fluorescein, and therefore the conditions of support, thrust, position and/or obstruction of the tongue.

### 3.4. Chewing

A clinical evaluation will be carried out by a speech therapist specialized in the area of Orofacial Motricity, to check for possible improvement in chewing. For this, the gnathodynamometer, test that allows to measure the force of the occlusion, will be used.

### 3.5. Speech

A clinical evaluation will be carried out by a speech therapist specialized in the area of Orofacial Motricity, to check for possible improvement in speech. For this, the Glatzel test will be used to measure the degree of nasal patency and the functional symmetry of the upper airways both at rest and in function. Specifically in the functional evaluation, its usefulness is linked to the verification of the existence of nasality or hyponasality before vowels elicited by the patient.

### 3.6. Presence of bruxism

The bruxism questionnaire will be delivered to parents or guardians, which will be completed prior to treatment and afterwards. The questionnaire contains 4 questions regarding the perceived presence of bruxism, and 5 questions for the dentist to answer, based on a clinical observation. All questions have the options “yes” or “no.” The more times the word “yes” is chosen, the greater the risk for bruxism is.

### 3.7. Sleep quality

A Pediatric Sleep Quality Questionnaire will be delivered to parents or guardians, and it will be completed prior to treatment and afterwards. The questionnaire contains 44 questions regarding sleep quality. All questions have the options “yes” or “no.” The more times the word “yes” is chosen, the worse is the sleep quality.

### 3.8. Statistical analysis

The data will be tabulated and processed with the software GraphPad PRISM version 7.0. The values will be tested for normality by the Kolmogorov–Smirnov test, and will be expressed as mean and standard deviation if Gauss curve is assumed. For the comparison between the groups, the *t* test will be carried out, considering a level of significance of 0.5% (*P* < .05). No loses greater than 5% of the sample are expected.

## 4. Discussion

The treatment of bruxism is a challenge faced in the clinical practice. Treatment methods that are commonly used for treating bruxism in pediatric patients are kinesiotherapy, massage, infrared therapy, and PBM. Among them, PBM is noninvasive, cost-effective and painless.^[20,21]^ A randomized controlled trial tested the effectiveness of PBM in acupuncture points as an alternative treatment for sleep bruxism. The researchers came to the conclusion that children with sleep bruxism responded well to PBM therapy, as evidenced by the reduction in bite force and reports of headache.^[22]^

A systematic review conducted in 2021 aimed to evaluate the efficacy of PBM in the treatment of myofascial pain associated with TMD, and to perform a cost-effectiveness analysis of TMD treatment with PBM in patients with myofascial pain. The authors came to the conclusion that laser-treated groups had painful symptoms improvement that was superior to the CG and PBM was more cost-effective than placebo in patients with TMD and myofascial pain.^[23]^

Physiotherapy is another therapy proposed for reducing bruxism in children. The “self-awareness of movement” concept was put forth to improve the organization and coordination of body movements. However, it was analyzed only by a few studies.^[21]^

Although local PBM, acupuncture PBM and physiotherapy have been studied in the treatment of bruxism, this is the first study to evaluate the effect of ILIB combined with myofunctional exercises for sleep bruxism in pediatrics.

## Author contributions

Conceive and design the study: NOV, SKB, MLLG, RAMF, KPSF, ACRTH; will perform the experiment: NOV, LHB, ANF, MRFS; will analyze the data: EMS, LJM; write the paper: NOV, APTS, MLLG, SKB.

**Conceptualization:** Natalia Osorio Viarengo, Raquel Agnelli Mesquita-Ferrari, Kristianne Porta Santos Fernandes, Anna Carolina Ratto Tempestini Horliana, Sandra Kalil Bussadori.

**Formal analysis:** Elaine Marcilio Santos, Lara Jansiski Motta.

**Investigation:** Natalia Osorio Viarengo, Laura Hermida Bruno, Ana Laura Fossati, María Roxana Ferreira Sertaje.

**Methodology:** Natalia Osorio Viarengo.

**Supervision:** Sandra Kalil Bussadori.

**Writing – original draft:** Natalia Osorio Viarengo.

**Writing – review & editing:** Marcela Leticia Leal Gonçalves, Ana Paula Taboada Sobral, Sandra Kalil Bussadori.
